# Molecular Systematics of Genus *Atractylodes* (Compositae, Cardueae): Evidence from Internal Transcribed Spacer (ITS) and trn*L*-*F* Sequences

**DOI:** 10.3390/ijms131114623

**Published:** 2012-11-09

**Authors:** Hua-Sheng Peng, Qing-Jun Yuan, Qian-Quan Li, Lu-Qi Huang

**Affiliations:** 1 Institute of Chinese Materia Medica, China Academy of Chinese Medical Science, Beijing 100700, China; E-Mails: penghuasheng19752@yahoo.com.cn (H.-S.P.); yuanqingjun@icmm.ac.cn (Q.-J.Y.); liqianquanmg@yahoo.cn (Q.-Q.L.); 2 Department of Pharmacy, Anhui University of Traditional Chinese Medicine, Hefei 230031, China

**Keywords:** *Atractylodes*, ITS, phylogenetics, plastid DNA, trn*L*-*F*

## Abstract

To determine the evolutionary relationships among all members of the genus *Atractylodes* (Compositae, Cardueae), we conducted molecular phylogenetic analyses of one nuclear DNA (nrDNA) region (internal transcribed spacer, ITS) and one chloroplast DNA (cpDNA) region (intergenic spacer region of trn*L*-*F*). In ITS and ITS + trn*L*-*F* trees, all members of *Atractylodes* form a monophyletic clade. *Atractylodes* is a sister group of the *Carlina* and *Atractylis* branch. *Atractylodes* species were distributed among three clades: (1) *A. carlinoides* (located in the lowest base of the *Atractylodes* phylogenetic tree), (2) *A. macrocephala*, and (3) the *A. lancea* complex, including *A. japonica*, *A. coreana*, *A. lancea*, *A. lancea* subsp. *luotianensis*, and *A. chinensis*. The taxonomic controversy over the classification of species of *Atractylodes* is mainly concentrated in the *A. lancea* complex. In base on molecular results, the intraspecific division of *Atractylodes lancea* is not supported, and *A. coreana* should be treated as a synonym *A. chinensis*.

## 1. Introduciton

*Atractylodes* is an important medicinal genus in East Asia. With over 2000 years of medicinal history, all of its species have been used as medicine except for *A. carlinoides*. Their rhizomes are generally referred to as “Zhu” in ancient herbal literature and are clinically divided into “Cangzhu” and “Baizhu” in Traditional Chinese Medicine [1]. In China, Japan, South Korea and North Korea, *Atractylodes* is a traditional medicine for treating gastroduodenal diseases.

Although *Atractylodes* was established by Candolle in 1838 [2], the relationship between *Atractylodes* plants and other related genera should be clarified. This genus was considered by some taxonomists as *Atractyli*s prior to its establishment [3–5]. After establishment of the genus, many taxonomists still placed it in *Atractylis*[3]. The sexuality of flowers is the main feature that distinguished *Atractylodes* from *Atractylis. Atractylodes* are dioecious and *Atractylis* are monoecious [5,6]. With further studies of numerous specimens and field observations, *Atractylodes* flowers were found to be either monoecious, as in *A. macrocephala* and *A. carlinoides*, or gynodioecious, as in *A. lancea*, *A. japonica* and *A. coreana*. The gynodioecism of *Atractylodes* can be attributed to the degeneration of stamens so that the monoecious flowers become pistillate. Sometimes the degeneration presents a continuous transition [7]. Based on morphologic observation, *Atractylodes lancea* and *A. japonica* are gynodioecious, whereas *A. macrocephala* is monoecious [7,8], similar to *Atractylis*. Based on its morphologic characteristics, Petit identified the closest relationship between *Atractylis* and *Atractylodes* and attributed two species (*Atractylodes comosa* and *Atractylodes cuneata*) in the Mediterranean area to *Atractylodes*[9]. Therefore, the relationship of *Atractylodes* with its related genera, *Atractylis*, should be examined.

On the other hand, the classification of interspecific and infraspecific taxa in *Atractylodes* is much debated [2–5]. As recorded in the “Flora Reipublicae Popularis Sinicae”, the genus has five species, namely, *Atractylodes macrocephala*, *A. lancea*, *A. carlinoides*, *A. japonica* and *A. coreana*[10–12]. However, as stipulated by the Chinese Pharmacopoeia, Cangzhu is derived from *Atractylodes lancea* and *A. chinensis* (a synonym of *A. lancea* in “Flora Reipublicae Popularis Sinicae”). In addition, the relationship between *A. corean*a and *A. chinensis* is controversial [13]. Hu *et al.*[14] published *Atractylodes lancea* subsp. *luotianensis*, whereas Zou *et al.*[13] suggested that *A. lancea* subsp. *luotianensis* should be included in *A. lancea* rather than be treated as a separate subspecies. However, as recorded in the “Flora of China” [15], *Atractylodes* comprises only four species: *Atractylodes carlinoides*, *A. macrocephala*, *A. coreana* and *A. lancea. A. japonica* was treated as a synonym of *A. lancea*. In fact, the rhizome of *Atractylodes japonica* is used as Cangzhu in China, and is used as Baizhu in Japanese Pharmacopoeia [16]. Controversies over the classification of species and infraspecific categories of *Atractylodes* affect the circulation and identification of medicinal materials. Hence, studies on the genetic relationships among *Atractylodes* species are conducive to understanding the relationship among different taxa, thereby confirming the classification of plants in this taxon.

The findings in molecular systematics in recent years, especially genealogy trees built using gene sequences, have provided concrete evidence for the phylogeny of Compositae [17,18]. Some scholars have already established the genetic relationship of *Atractylodes*, which, however, failed to cover all the taxa of *Atractylodes*[13,19,20]. The limited number of individuals in each taxon limits the available experimental materials; hence, the genealogy of *Atractylodes* is incomplete. In this research, internal transcribed spacer (ITS) and trn*L*-*F* were used to determine the genealogy of *Atractylodes* because ITS and trn*L*-*F* sequences have provided satisfying results in other taxa of Compositae [21–23]. Samples of all *Atractylodes* species, including the controversial species and subspecies, were collected. The main purpose of the present study aims to establish a complete molecular systematic genetic relationship of *Atractylodes* (1) to ascertain the systematic relationship between *Atractylodes* and *Atractylis* (2) to determine the genetic relationship among the different taxa of *Atractylodes*.

## 2. Results and Discussion

### 2.1. The Features of Alignments in cpDNA (trnL-F) and nrDNA (ITS)

The newly sequenced ITS and chloroplast trn*L*-*F* from 49 *Atractylodes* samples are obtained (GenBank accession numbers are given in the Appendix Table 1). The sequences characteristics and phylogenetic information generated by the chloroplast trn*L*-*F*, nuclear ITS, and the combined ITS + trn*L*-*F* sequences is given in Table 1.

### 2.2. The Relationship between Atractylodes and Related Genera

The results of the partition homogeneity test (ILD) was *p* = 0.10, which indicates that the two datasets (ITS and trnL-F) show no significant incongruence. We applied Maximum parsimony (MP), Maximum likelihood (ML), and Bayesian Inference (BI) methods to conduct the phylogenetic analysis of the ITS and trn*L*-*F* sequences.

**Figure 1:**
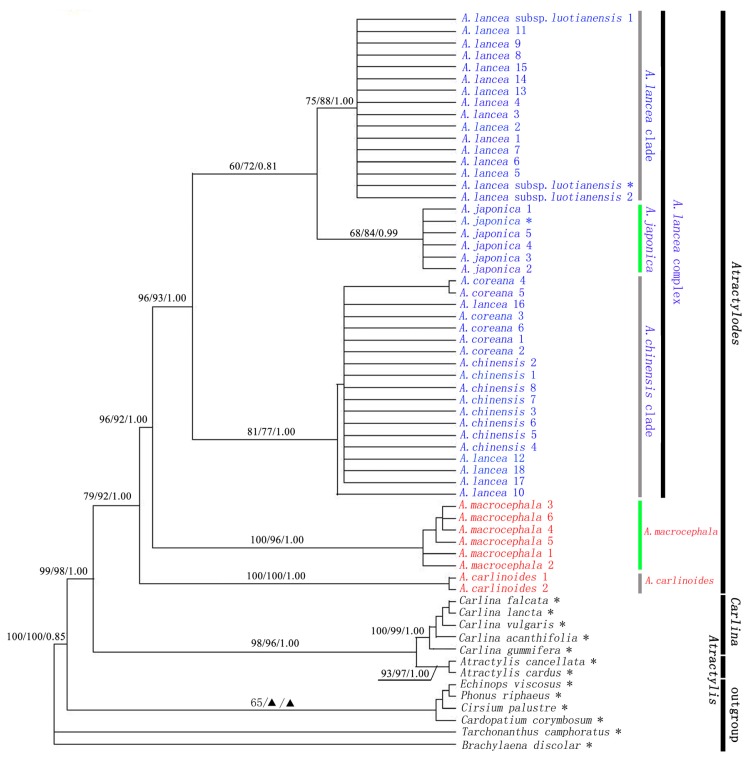
The 50% majority rule consensus tree from Bayesian Inference (BI) analysis of the combined ITS and trn*L*-*F* dataset; Maximum parsimony and ML bootstrap values and Bayesian posterior probabilities are shown above branches (MP/ML/BI). ▲ indicates ≤ 50% support. Taxa with * were downloaded from GenBank. Among the Atractylodes, the taxon with red color represent red flower, and the blue colored have white flower. Taxa with green color have obvious petiole, and gray colored have no petiole.

ITS and ITS + trn*L*-*F* phylogenetic trees, constructed based on three commonly used phylogenetic analysis methods (MP, ML, and BI) were very similar in terms of topologic structure, and only differed in bootstrap support, as shown in Figure 1,S1. The phylogenetic trees show that *Atractylis* and *Carlina* gathered into a clade, with higher bootstrap support rates (ITS + trn*L*-*F*: MP-BS/ML-BS/BI-PP = 98/96/1.00, Figure 1; ITS: MP-BS/ML-BS/BI-PP = 82/92/1.00, Figure S1; trn*L*-*F*: MP-BS/ML-BS/BI-PP = 83/95/1.00, Figure S2). *Atractylodes* is a sister group of the *Atractylis* and *Carlina* clade (ITS + trn*L*-*F*: MP-BS/ML-BS/BI-PP = 99/98/1.00, Figure 1; ITS: MP-BS/ML-BS/BI-PP = 85/96/1.00, Figure S1; trn*L*-*F*: MP-BS/ML-BS/BI-PP = 90/93/1.00, Figure S2). Although *Atractylodes* presents a relatively close genetic relationship with *Atractylis* in terms of morphologic characteristics [9,10]. However, in terms of biotypes and chemical composition, *Atractylodes* has obvious differences from *Atractylis. Atractylodes* is distributed in the Korean Peninsula, Japan, Russia, and other areas in Central China, in East Asia. *Atractylodes* is an endemic genus in the East Asia. *Atractylis* is geographically distributed in the Mediterranean area. The *Carlina* genus is distributed in the Canary Islands, the Mediterranean, and in temperate areas of Europe and Asia. *Carlina* is distributed in the Altay Area in Xinjiang Province, China, and is only represented by one species, *Carlina biebersteinii. Carlina* and *Atractylis* share a relatively close genetic relationship which is consistent with their geographic distribution.

### 2.3. Atractylodes is a Monophyletic Group

According to our phylogenetic analyses, all taxon of Atractylodes formed a well-supported clade, with high support rates (ITS + trn*L*-*F*: MP-BS/ML-BS/BI-PP = 79/92/1.00, Figure 1; ITS: MP-BS/ML-BS/BI-PP = 83/95/1.00, Figure S1; trn*L*-*F*: ML-BS = 0.96, Figure S2). It was shown that Atractylodes is a monophyletic group.

### 2.4. *Atractylodes carlinoides* is Sister Species of the Rest of *Atractylodes* Species

**Figure 2:**
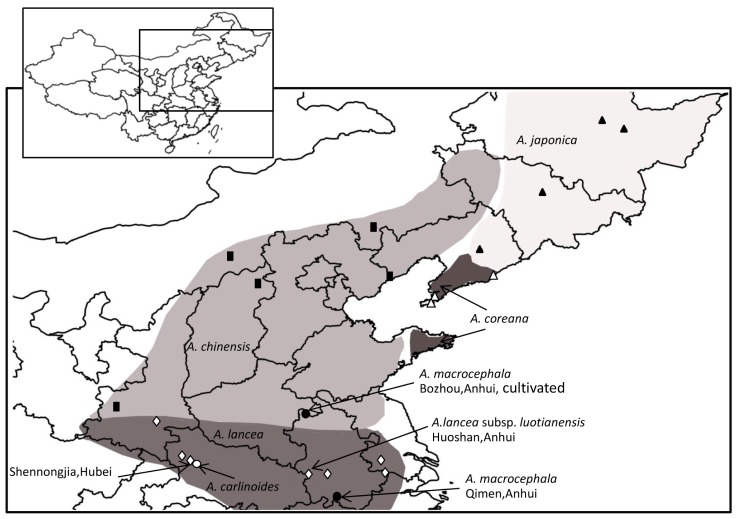
Geographic distribution of Atractylodes and sample collection location in China (▲: *A.japonica*; ■: *A.chinensis*; δ: *A. coreana*; ⋄: *A. lancea* or *A.lancea* subsp *luotianensis*; •: *A. macrocephala*;○: *A. carlinoides*).

*Atractylodes carlinoides* is endemic to Shennongjia and its vicinities (Figure 2). Previous studies were often lacking research due to the narrow distribution of *Atractylodes carlinoides*[19,20,24]. Morphologically, *Atractylodes carlinoides* presents significant differences from other *Atractylodes* species such as the presence of basal leaves in mature plants, the uniramous aerial stem, and the underdeveloped rhizome [6]. According to ITS and ITS + trn*L*-*F* phylogenetic trees, *Atractylodes* can be divided into three branches: *A. carlinoides*, *A. macrocephala*, and *A. lancea* complex. Among them, *Atractylodes carlinoides* is sister species of the rest of *Atractylodes* species, thus obtaining higher bootstrap support rates (ITS + trn*L*-*F* and ITS: MP-BS/ML-BS/BI-PP = 100/100/1.00, Figure 1,S1).

### 2.5. Genetic Relationship of the *Atractylodes lancea* Complex

According to ITS and ITS + trn*L*-*F* phylogenetic trees, *Atractylodes macrocephala* independently formed a branch as the sister group of the *A. lancea* complex, with high bootstrap support rates (ITS + trn*L*-*F*: MP-BS/ML-BS/BI-PP = 96/92/1.00, Figure 1; ITS: MP-BS/ML-BS/BI-PP = 90/88/0.98, Figure S1). *Atractylodes macrocephala* and *A. carlinoides* share some common aspects, including a large inflorescence diameter, red flowers, and monoecism. However, only *Atractylodes macrocephala* has pinnately divided leaves and plump, developed rhizomes. The common features of the *Atractylodes lancea* complex include smaller inflorescence diameters, white flower, and gynodioecism. In the phylogenetic tree, *Atractylodes macrocephala* is the sister group of the *A. lancea* complex, which is consistent with the findings of Zhou *et al.* and Mizukami *et al.*[13,19].

The taxonomic controversy over the classification of species and infraspecific taxa of *Atractylodes* is mainly concentrated in the *A. lancea* complex. The classification of the *Atractylodes lancea* complex is mainly based on leaf traits, such as petiole, divided leaves, and the degree of the division. Variation in these traits is related to geographical distribution and ecological environment, which leads to different classifications by scholars in terms of macrospecies or microspecies classification that ultimately generated masses of taxonomic controversy [6,25,26]. In our styudy, the *Atractylodes lancea* complex was divided into three subclades: *A. lancea* clade, *A. chinensis* clade, and *A. japonica* (Figure 1,S1).

*Atractylodes lancea* clade is mainly distributed in South China (such as in Anhui, Jiangsu, and Hubei Provinces), including *A. lancea* subsp. *luotianensis* and *A. lancea. A. lancea* subsp. *luotianensis* is a new geographic subspecies established by Hu [14], and it is found at altitudes above 600 m in the Dabie Mountain Area in Anhui and Hubei Province. The main difference of this subspecies from *Atractylodes lancea* is the larger diameter of its inflorescence. Continuous survey at different elevations of the Dabie Mountain area revealed that the traits of *A. lancea* continuously change with altitude [7]. In the present research, ITS analysis and the joint analysis of ITS and trn*L*-*F* demonstrate that *Atractylodes lancea* subsp. *luotianensis* shares a short genetic distance with *A. lancea*, which is consistent with the findings of Zou *et al.*[13]. Hence, the intraspecific division of *Atractylodes lancea* is not supported by our study.

*Atractylodes chinensis* clade includes *A. coreana*, *A. chinensis*, and a part of *A. lancea*. Whether *Atractylodes chinensis* should be identified as a species, a variant (*A. lancea* var. *chinensis*), or a synonym of *A. lancea* has been a controversial issue because *A. chinensis* is hard to distinguish from *A. lancea* in terms of biomorphology [6,10,25]. Shi [6,10] identified *Atractylodes lancea* as a polytypic species based on morphologic studies, and distinguishing *A. chinensis* from *A. lancea* is difficult based on traits such as leaf shape. Shi [6,10] also believed that *Atractylodes chinensis* should be a synonym of *A. lancea*. Scholars thought that *A. lancea* was in the south side of Qinlin Mountains, *A. chinensis* was in the north side, and there were some transitional types between the two species (Figure 2). Shiba *et al.*[24] attributed the continuous variation to hybrids of *Atractylodes lancea* and *A. chinensis*, which suggests that *A. lancea* and *A. chinensis* should be two independent species. However, in the phylogenetic tree, the *Atractylodes lancea* in Taibai and Zhen’an at the South Slope of Qinlin Mountains in Shaanxi Province are clustered with all *A. chinensis* and *A. coreana*. In the population from Shennongjia, two strains are clustered with *Atractylodes lancea* clade and one strain is clustered with *A. chinensis* clade. This finding indicates the inconsistent morphologic features and genetic differentiation between *Atractylodes lancea* and *A. chinensis*.

In “Flora Reipublicae Popularis Sinicae” and “Flora of China”, *Atractylodes coreana* was regarded as a separate species. *A. coreana* was only distributed in Liaodong and Shadong Peninsulas (Figure 2), and it have different characteristics such as lower and middle cauline leaves narrowly elliptic to ovate-lanceolate, undivided. However, in ITS and ITS + trn*L*-*F* phylogenetic trees, *Atractylodes coreana* and *A. chinensis* are clustered into one clade. Shiba *et al.*[24] indicated that all samples of *Atractylodes chinensis* and *A. coreana* have the same ITS genotype. This study show that *A. coreana* should be treated as a synonym

*Atractylodes japonica* was regarded as a separate species in “Flora Reipublicae Popularis Sinicae”. However, it was a synonym of *Atractylodes lancea* in “Flora of China”. According to ITS and ITS + trn*L*-*F* phylogenetic trees, *Atractylodes japonica* is clustered into its own branch among the *A. lancea* complex, and it is closely related to *A. lancea* clade. *Atractylodes japonica* have different morphological characters from the other taxa in *A. lancea* complex: the leaves have long petioles and are generally divided or completely divided into 3–5 lobes. The trnK phylogenetic tree revealed that *Atractylodes japonica* and *A. lancea* are most closely related [19]. Guo also indicated that *Atractylodes japonica* is most closely related to the *A. lancea* in Jiangsu Province while not that closely related to *A. chinensis* in Hebei, Beijing, and Shaanxi [27].

The phylogenetic trees show that *Atractylodes lancea* complex formed a monophyletic group, with higher support rate (ITS + trn*L*-*F*: MP-BS/ML-BS/BI-PP = 96/93/1.00, Figure1; ITS: MP-BS/ ML-BS/BI-PP = 88/90/1.00, Figure S1). However, three branches in the *Atractylodes lancea* complex (*A. lancea* clade, *A. chinensis* clade, and *A. japonica*) were lack of high support rate. Geographically, *Atractylodes lancea* and *A. chinensis* was distributed in the south and north side of Qinlin-Huaihe River each other. Morphologically, some specimens of *Atractylodes chinensis* or *A. lancea* in transitional areas are hard to clarify. In the phylogenetic tree, some samples of the *A. lancea* are clustered in *A. chinensis* clade. In the population from Shennongjia, two strains are clustered with *Atractylodes lancea* clade and one strain is clustered with *A. chinensis* clade. This finding indicates that geographical distribution, morphologic features and genetic differentiation of *Atractylodes lancea* clade and *A. chinensis* clade are not entirely consistent. For a profound understanding of the relationship between the genetic differentiation of *Atractylodes* and its geographic distribution, an in-depth investigation into the subject with molecular phylogeography is needed.

## 3. Experimental Section

### 3.1. Selection of Taxa and Outgroups

Samples were collected from related producing areas according to voucher specimens or literature based on the view of microspecies, because the classification of species and infraspecific taxa of *Atractylodes* is much debated (The areas where the samples are produced are shown in Figure 2 and Table S1) [5,6,10,26]. The tested materials were fresh leaves. After collection, the samples were immediately dried in silica gel for DNA extraction and sequence analysis. After identification by the first author, the samples were preserved and deposited with the Institute of Chinese Materia Medica, China Academy of Chinese Medical Sciences.

*Atractlis*, *Carlina*, and *Atractylodes* belong to subtribe Carlininae [28]. To study the relationship of *Atractylodes* with *Atractlis*, and *Carlina*, the genera *Brachylaena*, *Cardopatium*, *Cirsium*, *Echinops*, *Phonus* and *Tarchonanthus* were used as outgroups in the phylogenetic analyses based on the studies of Garcia-Jacas *et al.*[28] and Susanna *et al.*[17]. Their ITS and trn*L*-*F* sequences were downloaded from GenBank (GenBank accession numbers are shown in Table S2).

### 3.2. DNA Extraction, Amplification, and Sequencing

The cetyltrimethylammonium bromide method [29] was used to extract genomic DNA from the silica gel-dried leaves. The chloroplast DNA sequences were amplified using trn*L* as the forward primer and trn*F* as the reverse primer [30]. The nuclear ribosomal sequence was amplified using ITS5 as the forward primer and ITS4 as the reverse primer [31]. All sequences used 20 μL PCR reaction contained 14.7 μL of ddH_2_O, 2 μL of 10× buffer (TaKaRa Biotech, Dalian, Beijing, China), 1.6 μL of dNTP (TaKaRa Biotech, Dalian, Beijing, China), 0.5 μL of each primer, 0.5 μL of template DNA (1 μg), and 0.2 μL of Ex Taq DNA polymerase (TaKaRa Biotech, Dalian, Beijing, China). The PCR conditions were as follows: initial denaturation at 95 °C for 5 min, followed by 35 cycles of 95 °C for 1 min, 54 °C (ITS) or 58 °C (trn*L*-*F*) for 30 s, 72 °C for 1 min 40 s, and final extension at 72 °C for 5 min. The PCR products were run on 1.0% agarose gel in 1.0× Tris-borate-EDTA buffer, purified using a Tiangen Midi Purification Kit (Tiangen Biotech, Beijing, China), and then sequenced using a Bigdye Terminator Cycles Sequencing Ready Reaction Kit and Applied Biosystems ABI3730 DNA Sequencer.

### 3.3. Sequence Alignment and Phylogenetic Analysis

Susanna *et al.*[17] used ITS, trn*L*-*F*, and matK to study the phylogenetic relationship of Cardueae. ITS, trn*L*-*F*, and matK sequence and analysis was also applied in the present study. However, our studies show that the substitution sites of matK in *Atractylodes* are very limited; thus, ITS and trn*L*-*F* were reported.

The raw sequences were assembled and edited using the BioEdit software ver.5.0.9 [32] and adjusted manually as necessary. The novel sequences generated from *Atractylodes* and the sequences downloaded from GenBank were subjected to multiple alignment using the CLUSTALW 1.83 software package with default settings [33]. ITS and trn*L*-*F* + ITS datasets were constructed, and all contained 49 individuals from 7 taxa.

To examine the extent of conflict between the ITS and trn*L*-*F* datasets, the partition homogeneity test (also known as the incongruence length difference, ILD; [34]) was carried out using PAUP*. The text was implemented with 1000 partition homogeneity test replicates, using a heuristic search option with simple addition of taxa, TBR branch swapping and MaxTrees ser to 1000. The results suggest that both data sets are congruent (*p*-value > 0.05) and can be combined.

Parsimony analysis. Maximum parsimony (MP) analyses were carried out using PAUP* v.4.0b10 [35]. For each analysis, maximum parsimony trees were sought using the heuristic search strategies of PAUP* (with 1000 replicate analyses, random stepwise addition of taxa, tree bisection and reconnection (TBR) branch swapping, and setting the maximum number of trees to 10,000). Bootstrap values were calculated from 1,000,000 replicate analyses using fast stepwise addition of taxa, and only those values compatible with the majority-rule consensus tree were recorded. All most parsimonious trees (MPTs) were saved and PAUP* was used to compute a strict consensus. Tree lengths, consistency index (CI) and retention index (RI) were calculated excluding uninformative characters.

Bayesian Inference (BI) analysis. Datasets were conducted using MrBayes version 3.1.2 [36]. Prior to these analyses, the best-fit nucleotide substitution models of each partition were selected by AIC implemented in MrModeltest v.2.3 [37] (GTR + G for the ITS datasets, GTR for the cpDNA datasets, GTR + I + G for the cpDNA +nrDNA datasets). Four simultaneous Markov chain Monte Carlo (MCMC) chains were run using MrBayes version 3.1.2 [38], there heated and one cold, with the temperature adjusted to 0.5 in order to keep an appropriate heat range for the four chains. From a random starting tree, the Bayesian analysis was run for 20 million generations, and the trees were saved to a file every 1000 generations. Branch lengths of the trees were saved. Each analysis reached stationarity (the average standard deviation of split frequencies between runs ≤0.01) well before the end of the run. Burn-in (=5,000) trees were discarded, and the remaining trees and their parameters were saved. The 50% majority rule consensus tree was constructed. The results of the Bayesian analysis are reported as posterior probabilities [38], which are equal to the percentage of trees sampled when a given clade is resolved. Only PP scores exceeding 50% are shown.

Maximum likelihood (ML) analysis. ML analyses were performed using PHYML version 2.4 [39] using the same model of substitution with 1000 bootstrap replicates.

## 4. Conclusions

In *Atractylodes*, the ITS region from the nuclear genome was more variable than the *trn*L-*trn*F region from the plastid genome. All members of *Atractylodes* formed a well-supported clade, which is a sister group of the *Carlina* and *Atractylis. Atractylodes* species were distributed among three clades: *A. carlinoides*, *A. macrocephala* and the *A. lancea* complex (including *A. japonica*, *A. coreana*, *A. lancea*, *A. lancea* subsp. *luotianensis*, and *A. chinensis*). *Atractylodes carlinoides* is a sister species of the rest of the *Atractylodes* species. The taxonomic controversy over the classification of species of *Atractylodes* is mainly concentrated on the *A. lancea* complex*. A. lancea* subsp. *luotianensis* shares a short genetic distance with *A. lancea*; therefore, it should be treated as a synonym of *A. lancea*. Based on molecular results, *Atractylodes coreana* should be treated as a synonym of *A. chinensis.*

## Figures and Tables

**Table 1:** Comparison of Phylogenetic Information for *Atractylodes* Species from internal transcribed spacer (ITS), trn*L*-*F*, and combines data sets.

Parameter	ITS	trn*L*-*F*	Combines
Number of accession	49	49	49
Range of sequence length (bp)	642–643	799–801	1442–1443
Characters of data matrix	643	800	1443
Number of constant sites (%)	584 (90.82%)	793 (99.13%)	1377 (96.09%)
Number of parsimony-information sites (%)	55 (8.55%)	7 (0.88%)	62 (4.32%)
Number of autopomorphic-information sites (%)	4 (0.62%)	0 (0.00%)	4 (0.28%)
Consistency index (CI)	0.892	0.969	0.891
Retention index (RI)	0.955	0.958	0.947
Rescaled consistency index (RC)	0.852	0.928	0.844
Homoplasy index (HI)	0.108	0.031	0.109
